# Wickersheimer’s Preparation

**Published:** 1881-02

**Authors:** 


### WICKERSHEIMER'S PREPARATION.

THE following formulæ, according to the Boston Journal of Chemistry, is now adopted by the manufacturers of the Wickersheimer preserving fluid:

| | For Injecting. | For Immersing. |
|---|---|---|
| Arsenious acid, | 16 grammes, | 12 grammes. |
| Sodium chloride, | 80 " | 60 " |
| Potassium sulphate, | 200 " | 150 " |
| " nitrate, | 25 " | 18 " |
| " carbonate | 10 " | 15 " |
| Water, | 20 litres, | 10 " |
| Glycerine, | 4 " | 4 " |
| Wood naphtha, | ¾ " | ¾ " |

Hager suggests the following as a substitute for Wickersheimer's preparation:

| | |
|---|---|
| Salicylic acid, | 4 drachms, |
| Boracic acid, | 5 " |
| Potassium carbonate, | 1 " |
| Dissolved in hot water, | 12½ ounces, |
| Glycerine | 5 " |

Then add oil cinnamon, oil cloves, each 3 drachms, dissolved in alcohol, 12½ ounces. The latter fluid is not poisonous, and possesses the desirable property of acting as an antiseptic, and is possessed of a pleasant odor.

